# Surface Structuring by Laser Remelting (WaveShape): Microstructuring of Ti6Al4V for a Small Laser Beam Diameter and High Scan Speeds

**DOI:** 10.3390/mi12060660

**Published:** 2021-06-03

**Authors:** André Temmler, Shan Qi

**Affiliations:** 1 Fraunhofer Institute for Applied Optics and Precision Engineering (IOF), Albert-Einstein-Straße 7, 07745 Jena, Germany; 2 Chair for Laser Technology, RWTH Aachen University, Steinbachstraße 15, 52074 Aachen, Germany; shan.qi@hotmail.com

**Keywords:** laser remelting, titanium Ti6Al4V, Waveshape, surface topography, surface engineering

## Abstract

The appearance of a surface is a crucial characteristic of a part or component. Laser-based micromachining gets increasingly important in generating tailored surface topographies. A novel structuring technique for surface engineering is surface structuring by laser remelting (WaveShape), in which surface features are created without material loss. In this study, we investigated the evolution of surface topographies on Ti6Al4V for a laser beam diameter of 50 µm and scan speeds larger than 100 mm/s. Surface features with aspect ratios (ratio of height to width) of almost 1:1 were achieved using the WaveShape process. Furthermore, wavelengths smaller than 500 µm could be effectively structured using scan speeds of up to 500 mm/s. The experimental results showed further that the efficiency of the WaveShape process in terms of achieved structure height per unit time significantly increases for high scan speeds.

## 1. Introduction

Surface structuring processes have long been established for industrial manufacturing processes. As a result of the manufacturing processes, surfaces are often designed and produced according to specific customer requirements. The development of structuring techniques requires, among other things, miniaturization of surface structures in the respective field of application. In general, mechanical tools are often used for surface processing. In the field of micro- and nanomachining of surfaces, however, the use of tools is only possible to a limited extent. Therefore, contactless, laser-based surface structuring techniques utilizing small interaction areas are becoming increasingly important.

Surface structuring in laser beam-based processes is often achieved by partial vaporization of the surface. Chichkov et al. [1] laid a basis for a deepened understanding of (ultra) short pulsed laser ablation of solids. Since then, hundreds of studies have been conducted to further investigate the specific interdependencies, characteristics, and outcomes for laser ablation processes on all kinds of materials. A recent example for ns-ablation of metals is the study of Zhang et al. [2], in which a good agreement was achieved between simulation and experiments for laser ablation of stainless steel. Additionally, laser ablation by ultra-short pulses is a topic of high scientific and potentially industrial relevance. For example, Finger et al. [3] showed that high-precision surface modifications are feasible without excessively increasing surface roughness using fs laser pulses. Nonetheless, laser ablation is still a subtractive method in this regard similar to traditional mechanical techniques and material is lost during processing. Alternatives to subtractive methods are energy beam processes, which achieve a redistribution of molten material at the surface. Currently, there are particularly three different processes of high scientific and industrial relevance, which achieve a material redistribution in the molten phase: DLIP, SurfiSculpt, and WaveShape.

Direct laser interference patterning (DLIP) results from two or more coherent laser beams interfering at a workpiece surface [4] (for ns-pulses [5] or for ps-pulses [6]). The superposition of coherent laser beams creates a defined interference pattern, which is directly transferred/imprinted on a surface by a variety of different processes, e.g., photo-physical, photo-thermal or photo-chemical processes [7]. Since the interference patterns often show a periodicity in the submicrometer or micrometer range, DLIP typically creates surface features with sizes in the same order of magnitude [5]. This process seems to be particularly applicable on surfaces with a low surface roughness, since the periodicity and size of features are typically in the micrometer range (e.g., Ra < 100 nm) [8]. Alternatively, the creation of hierarchical, periodical structures are a feasible and promising way to combine surface features in the millimeter range with periodic patterns from the DLIP process [9]. Specifically, the combination of direct laser writing (DLW) and DLIP was already investigated to successfully create these kinds of hierarchical, periodical surface features on Ti6Al4V samples [10]. The fundamental mechanisms of DLIP are thought to be the redistribution of molten material. The melt pool dynamics as a result of thermal gradients induced by the interference pattern at the work piece surface are assumed to be the main physical effect for surface structure formation [11]. In terms of processing speed, Lasagni et al. [12] showed that area rates of up to 60 cm^2^/s (or approx. 167 s/m^2^) are already feasible on stainless steel. Overall, DLIP is a promising surface texturing technique, but also has its limitations, e.g., in terms of achievable dimensions of the generated surface features.

Initially, the Surfi-Sculpt© process primarily utilized an electron beam to create surface features from a molten state. Dance and Buxton [13] introduced this technique and the TWI (The Welding Institute, GB) patented the Surfi-Sculpt© process. The process is best known for its characteristic surface features, since it is primarily used to generate spikes with a large aspect ratio of height to width [14]. Blackburn and Hilton [15] showcased that not only an electron beam could be used for Surfi-Sculpt© but also a laser beam source, e.g., a fiber laser. The authors already demonstrated that surface features could be created on a broad variety of different materials such as stainless steel, titanium alloy Ti6Al4V, and nickel-based superalloy Inconel 718. Furthermore, Buxton et al. [16] showcased that the process is not limited to metals but can also be used for glasses, ceramics, and polymers. In terms of the theoretical understanding and modeling of the process, Earl et al. [17] found indications that laser induced thermocapillary flow is a main reason for surface structure formation. Therefore, melt pool inherent material flow is thought to be mainly responsible for the redistribution of the molten material. Additionally, since a keyhole is thought to be created in the process, vapor pressure is assumed to play another key role in the formation of surface features [18]. Particularly, the keyhole dynamics lead to partly unsolved challenges in the prediction of the exact dimension of the resulting surface features generated in the Surfi-Sculpt© process [19]. Typical drawbacks of Surfi-Scuplt are the resulting surface roughness, the singular character of the surface features, and the partly unpredictable feature dimensions. 

Surface structuring by laser remelting (WaveShape) is a novel surface structuring process used for structuring metallic surfaces in the micro- and millimeter range by power modulation of a laser beam source. In contrast to other laser structuring processes, surface features are not generated by localized ablation but created by redistribution of molten material at the surface [20,21]. An attempt for modeling of the WaveShape process of Ti6Al4V was presented by Sharma et al. [22]. Although their model showed already some surprisingly good agreement with experimental results [23], Temmler and Pirch [24] pointed out some deficiencies of this model and introduced an overhauled, enhanced model. This model not only showed a good agreement with experimental results on tool steel H11, but also could be further improved (implementing effects from vapor pressure) to show an even better agreement with experimental results for the WaveShape process on Inconel 718 [25]. Arnaud et al. [26] could further expand the range or processable materials to a variety of steels and AlMg alloys. Since the surface roughness of the workpiece is simultaneously smoothed during structuring, Bordatchev et al. [27] could showcase a promising application of the WaveShape process for light guiding structures in lighting and automotive applications. Overall, the WaveShape process closes a gap for structures in the micro- and millimeter range and can achieve predictable, periodic, and aperiodic structures with heights from the micrometer to the millimeter range, while reducing surface roughness at the same time. However, the minimum size of the achievable structures is limited by the laser beam diameter and the dynamics of the laser beam source [28].

This study significantly extends previous investigations on the WaveShape process for small laser beam diameters (*d_L_* = 50 µm) and high processing speeds (*v_scan_* ≥ 100 mm/s) on Ti6Al4V. The foci of this study are to achieve significantly smaller wavelengths, to reduce processing time, and to increase process efficiency. Furthermore, high aspect ratios shall be achieved by high-speed multi-processing. The titanium alloy Ti6Al4V was chosen since it has a wide range of industrial applications, particularly for aviation, aerospace, and medical engineering. Additionally, Ti6Al4V proved to be well suited for laser remelting processes in general and for WaveShape in particular [23].

## 2. Materials and Methods

### 2.1. Opto-Mechanical Setup

**Figure 1:**
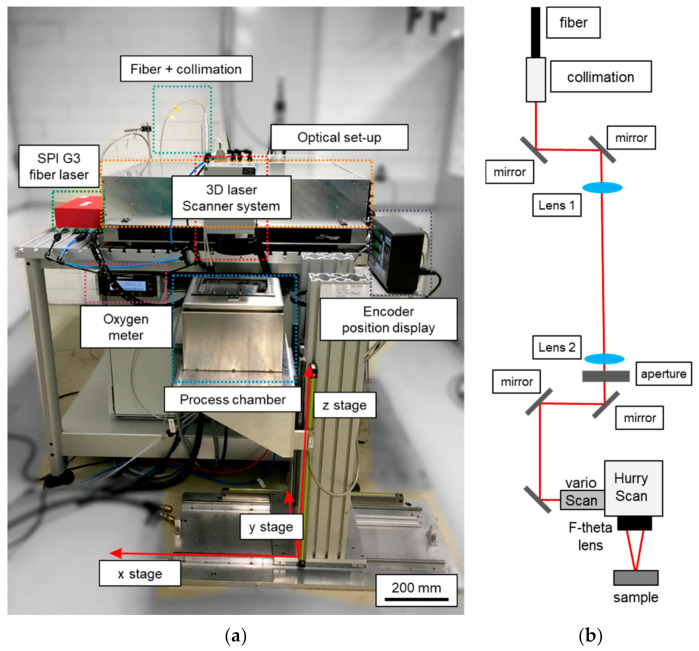
(**a**) Experimental setup, and (**b**) schematic of laser beam path and optical elements.

The “FluidStruc” laboratory system (Figure 1a) was used, which contains an end-pumped SPI fiber laser (SPI G3, 40 W, *λ_emi_*_t_ = 1062 ± 3 nm).

**Figure 2:**
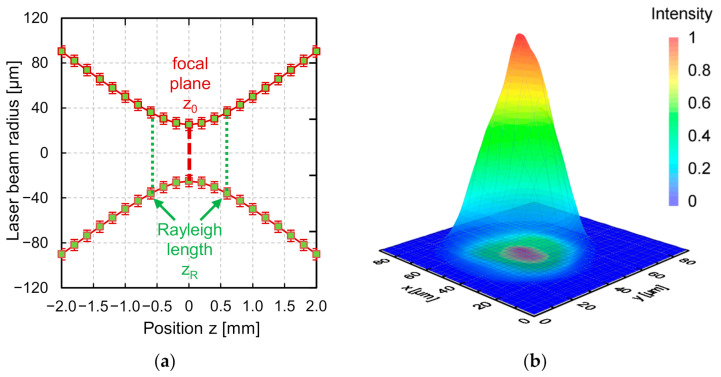
(**a**) Laser beam radius as function of distance to the focal lens (close to the focal plane), and (**b**) 3D representation of intensity distribution of laser beam in focal plane (*d_L_* = 50 µm).

This laser beam source emits multimodal laser radiation typically at a beam quality of *M*^2^ = 2.8–3.6. In this study, laser radiation was used in continuous wave (cw) mode with a maximum laser output power of approx. *P_L_* = 36 W at the workpiece surface. The laser beam was coupled into the optical setup (breadboard + optical elements) via an optical fiber and collimation with a collimated raw beam diameter of *r_Laser_* = 5.5 mm. Using various combinations of fused silica lenses and f-theta objectives, enables a wide range of different laser beam diameters on the workpiece for laser processing. In this setup, the lenses were used as a relay system in an 1:1 imaging configuration and an f-theta objective with a focal length of *f_T_* = 163 mm (Figure 1b) was used for focusing the laser beam. Laser processing took place in a process chamber, in which particularly the oxygen content was monitored and controlled. The resulting laser beam caustic and intensity distribution in the focal plane were measured for this investigation (Figure 2).

The measured beam characteristics (MicroSpotMonitor, Primes GmbH, Pfungstadt, Germany) were as follows: *M*^2^ = 3.2, Rayleigh length *z_R_* = 0.58 mm, depth of focus *DoF* (5%) = 0.19 mm, beam divergence *Θ_f_* = 87 mrad, and laser beam diameter *d_L_* = 50.0 (±2.4) µm (86% energy inclusion).

### 2.2. Material and Sample Preparation

The material investigated in this work was the titanium alloy Ti6Al4V (material no. 3.7165) which has very high strength and corrosion resistance. Ti6Al4V is categorized as light metal due to the low density and is often used in aerospace and medical applications. Table 1 shows a tabular overview of the elementary composition of Ti6Al4V.

Cylindrical round specimens made of Ti6Al4V and titanium are used to perform the experimental investigations. The diameter *d_Sample_* of the round samples is 80 ± 1.0 mm, while the thickness *t_Sample_* is 18 ± 0.5 mm. The initial surface was remelted, milled, and ground to a homogenous surface roughness of Sa = 0.31 ± 0.04 µm.

### 2.3. WaveShape Process

**Figure 3:**
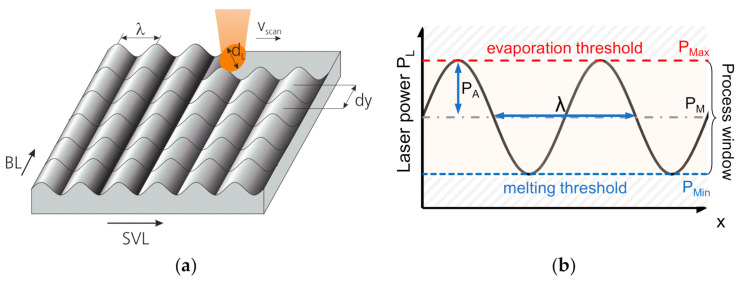
(**a**) Schematic for process strategy and crucial process parameters in WaveShape, and (**b**) schematic for sinusoidal modulation of laser power and process thresholds.

The active principle of surface structuring by laser remelting (WaveShape) is based on a remelting process of a surface boundary layer of a metal surface, while simultaneously modulating laser power in a controlled manner. Since the laser power is limited by melting and ablation thresholds, typically no material is removed during the process (Figure 3b). The controlled modulation of laser power (often sinusoidally) results in a continuous variation of the melt pool volume, which subsequently leads to formation of structure features such as periodic surface waves (Figure 3a). Since the surface solidifies from a molten state, the generated surface features typically show a small (micro-)roughness, which allows combining surface structuring and laser polishing in one process step. The active principle is explained in more detail in Temmler et al. [24].

In the WaveShape process, structuring is carried out by means of a remelting process that avoids material evaporation. The material is in a molten state during structuring. In a preliminary investigation, suitable process windows were determined for each combination of laser beam diameter and scan speed. This process window consists of an upper limit (*P_max_*) and a lower limit (*P_min_*), which span the process window between which the laser power was modulated during surface structuring (Equation (1) and Figure 3b):

(1) $$P_{L}\left(x\right)=P_{M}+P_{A}\cdot \sin\left(\;\frac{x}{\lambda }\cdot 2\pi \;\right).$$

In addition, the determined average laser power *P_M_* and maximal laser power amplitude *P_A,max_* were adjusted to the respective scan speed in order to ensure that the tests were always carried out within the process limits. The linear fit is done arithmetically with a straight-line function. The resulting process parameters and range are shown in Table 2.

To avoid unwanted oxidation of the remelted surface, laser processing took place in a sealed process chamber and a residual oxygen content of less than 100 ppm was used in all experiments. Process or test fields each had the shape of a square with a side length of 5 mm. The side length in the machining direction is called scan vector length (SVL), which corresponds to the length of a single track. The individual tracks generated in a processing field were each processed unidirectionally.

An overview of the essential process parameters and their range of investigation is shown in Table 2. The track offset *dy* is the distance between the individual tracks. To avoid interfering heat effects of neighboring tracks, the track offset was set to 0.5 mm. The Scan speed *v_scan_* was investigated between 100 and 500 mm/s. The wavelength of laser power modulation was investigated between two times (2·*d_L_*) and eight times (8·*d_L_*) the laser beam diameter *d_L_*, since the most significant effects were expected in this range. The number of passes *n* were mainly investigated in the range from 1 to 32, since more repetitions lead to structure characteristics (height to width ratio), which made it impossible to be comprehensively measured by WLI.

### 2.4. Surface Analysis

The surface analysis is based on white light interferometry (WLI) images of the remelted tracks. Based on a longitudinal section along the middle of a remelted track a fast Fourier transformation was conducted to calculate the dominant spatial frequency and the corresponding amplitude. The dominant spatial frequency corresponds to the structuring wavelength *λ* and the amplitude corresponds to structure height *h*. At least four single tracks of 5 mm length were used to determine the structure height. The specifics of this method are described in detail in various publications [20,23,24,25,28].

## 3. Results

### 3.1. Track Width

**Figure 4:**
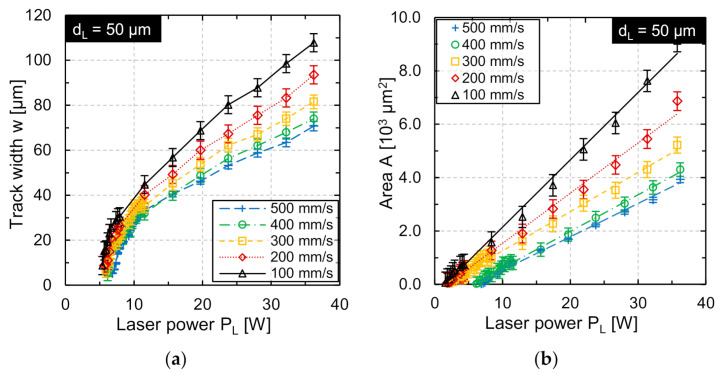
(**a**) Width of the remelted track as function of laser power and scan speed; (**b**) magnification for laser power *P_L_* = 8–12 W; and (**c**) squared track width as function of laser power (*d_L_* = 50 µm).

At first, the track width was determined as function of laser power and scan speed. This was helpful when an appropriate process window and a suitable track offset for areal structuring needed to be determined. For each set of laser power and scan speed, five single tracks with a length of 10 mm and a track offset of *dy* = 1 mm were structured and analyzed by microscopy (Figure 4a).

Typically, minimal laser power is not chosen to be directly at the melting threshold, but a little bit higher, so that the effective width of the remelted track is approximately half of the nominal laser beam diameter. This ensures that no discontinuous remelting occurs, which could negatively affect the continuity of the sinusoidal track profile [24,29]. Figure 4b shows that the remelted area (roughly approximated by the squared track width) is approximately a linear function of laser power, which indicates a 1D heat conduction condition (track width >> melt depth) similar to pulsed laser processing [30].

### 3.2. Laser Power Amplitude

Previous work of Bordatchev et al. [27] and Temmler et al. [28] demonstrated that the laser power amplitude typically has a significant effect on the obtained structure height and was therefore investigated first. The laser power amplitude *P_A_* was varied in five equidistant steps. The investigation was carried out for a laser beam diameter of *d_L_* = 50 µm and for scan speeds of *v_scan_* = 100 to 500 mm/s. The maximum laser power amplitude was deliberately exceeded by 25%, resulting in material vaporization and discontinuous processing. This was done to ensure that the process parameters were within reasonable limits. In this case, the lower or upper laser power limits for the laser power amplitude were specifically undershot or exceeded, respectively. As an example, the achieved structure heights *h* are shown as function of the varied laser power amplitude *P_A_* and the wavelength *λ* at constant scan speed *v_scan_* = 200 mm/s. The wavelength was investigated in the range *λ* = 0.1 to 0.4 mm (in equidistant steps of 0.1 mm).

**Figure 5:**
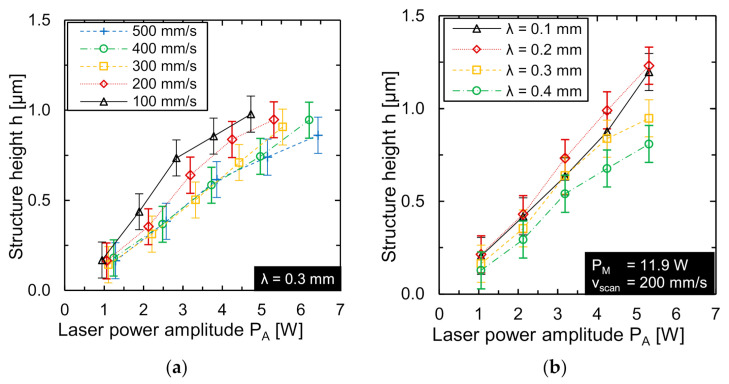
(**a**) Structure height *h* as function of laser power amplitude *P_A_* for different scan speeds (*d_L_* = 50 µm, λ = 0.3 mm, *P_M_* and *P_A_* adapted to *v_scan_*), and (**b**) structure height as function of laser power amplitude for different wavelengths (*d_L_* = 50 µm, *P_M_* = 12.2 W, *v_scan_* = 200 mm/s).

**Figure 6:**
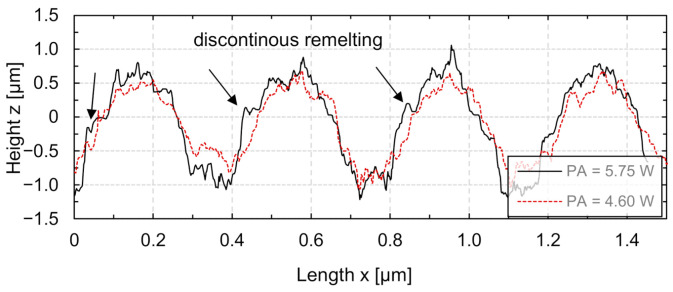
Longitudinal sections of single tracks after surface structuring using *P_A_* = 5.75 W and *P_A_* = 4.6 W (*d_L_* = 50 µm, *v_scan_* = 200 mm/s, *λ* = 0.2 mm, *P_M_* = 12.2 W).

Figure 5 shows that the structure height *h* increases linearly as function of laser power amplitude. For example, the structure height obtained at a wavelength *λ* = 0.1 mm was approx. 0.19 µm at *P_A_* = 1.15 W and increased almost linearly to approx. 0.96 µm at *P_A_* = 5.75 W. Compared to the structures generated at other wavelengths, the structure height obtained was greatest at a wavelength *λ* = 0.2 mm (four times the laser beam diameter). Temmler et al. [24] showcased that exceeding the determined maximum laser power amplitude leads to a deviation of the structure cross-section from a sinusoidal shape. With regards to the continuity of the remelting process, longitudinal sections of the generated structures at *v_scan_* = 200 mm/s and wavelength *λ* = 0.2 mm (four times the laser beam diameter) are shown in Figure 6.

Continuous remelting no longer occurred at a laser power amplitude of *P_A_* = 5.75 W and led to an additional deviation from a sinusoidal longitudinal section. The shape deviation shown in Figure 6 at *P_A_* = 5.75 W occurred since the laser power fell below the minimum laser power limit *P_min_*. In this case, no material was molten for a short time. Subsequently, the melt pool was formed again when the laser power was within the process limits again. However, this led to a significant shape deviation of the structure profile from an ideal sinusoidal form. Furthermore, this shape deviation was only observed at larger wavelengths (*λ* ≥ 0.2 mm). In addition, a pronounced high-frequency structural noise was observed on the resulting surface topography. Due to the small laser beam diameter, a comparatively small amount of material was remelted during processing. Thus, the initial surface roughness had a larger influence on the resulting surface topography.

### 3.3. Wavelength

**Figure 7:**
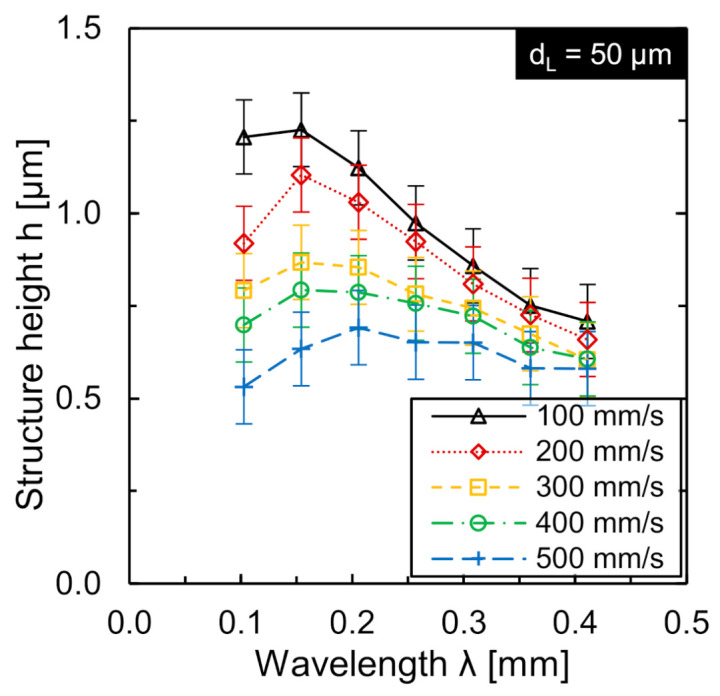
Structure height *h* as function of normalized wavelength for five different scan speeds *v_scan_* (*d_L_* = 50 µm, *P_A_* and *P_M_* linearly adapted to *v_scan_*).

The laser power amplitude *P_A_* and wavelength *λ* determine the maximal laser power gradient. At constant laser power amplitude, the wavelength has a significant influence on the local laser power gradient and hence temperature gradient and was therefore investigated in detail. The wavelength was investigated as multiples of the laser beam diameter (*λ* = 0.1 mm to *λ* = 0.8 mm, in 0.05 mm steps) and scan speeds *v_scan_* (*v_scan_* = 100 mm/s to *v_scan_* = 500 mm/s; in 100 mm/s steps). The achieved structure heights as function of wavelength for different scan speeds are shown in Figure 7.

At a scan speed of *v_scan_* = 100 mm/s, the maximum structure height *h* was approx. 1.23 µm at a wavelength of *λ* = 0.15 mm. The respective structure height achieved at twice and three times the laser beam diameter was approx. 0.84 and 0.9 µm, respectively. At *v_scan_* = 200 mm/s, the maximum structure height was *h* = 1.1 µm, also at a wavelength of *λ* = 0.15 mm. The structure height is *h* = 0.92 µm at *λ* = 0.1 mm and *h* = 1.03 µm at *λ* = 0.2 mm. When using even larger scan speeds from *v_scan_* = 300 to 500 mm/s, structuring at small wavelengths (*λ* = 0.1 mm or 0.15 mm) only occurred with a reduced laser power amplitude, since technical limits in terms of the maximal modulation frequency of laser beam source or control card were exceeded. The maximum structure height at a scan speed of *v_scan_* = 300 mm/s was generated at the wavelength *λ* = 0.15 mm and is approx. 0.87 µm. The maximum structure height is achieved at wavelength *λ* = 0.2 mm for scan speeds of *v_scan_* = 400 mm/s (*h* = 0.79 µm) and *v_scan_* = 500 mm/s (and *h* = 0.69 µm). In general, the achieved structure height became smaller as the scan speed increased, even though the laser power was adapted to the scan speed and laser beam diameter (Figure 7).

**Figure 8:**
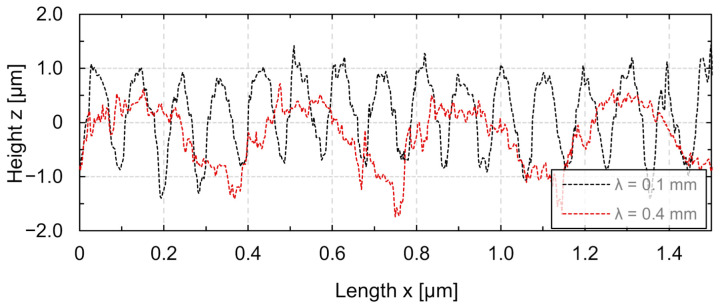
Representative longitudinal sections along the middle of the remelted tracks for λ = 0.1 mm and *λ* = 0.4 mm (*d_L_* = µm, *v_scan_* = 200 mm s^−1^, *P_M_* = 12.2 W, *P_A_* = 4.6 W).

Figure 8 shows longitudinal sections of single tracks for *v_scan_* = 200 mm/s at a wavelength of *λ* = 0.1 mm and *λ* = 0.4 mm, respectively. In general, the structure profile was only slightly skewed at the wavelength where the maximum structure height was achieved. The achieved structures were skewed in or against the scan direction when wavelengths were significantly larger and smaller, respectively, than the wavelength at which the maximum structure height was achieved (*λ* = 0.15–0.2 mm). Thus, the shape was deviating from the ideal sinusoidal shape along a longitudinal section.

### 3.4. Number of Repetitions

The investigations to this point have been carried out exclusively on the basis of single processing. A consequence is that only comparatively small structure heights were achieved so far (using large scan speeds and small laser beam diameters in the correspondingly adapted process window). Maximum structure heights in single track processing were obtained at wavelengths in the range *λ* = 0.15–0.25 mm for scan speeds from 100 to 500 mm/s. The maximum structure height *h_max_* = 1.23 µm was generated at *v_scan_* = 100 mm/s and *λ* = 0.15 mm. However, previous studies often showed that a significant increase in structure height was obtained by multiple processing.

**Figure 9:**
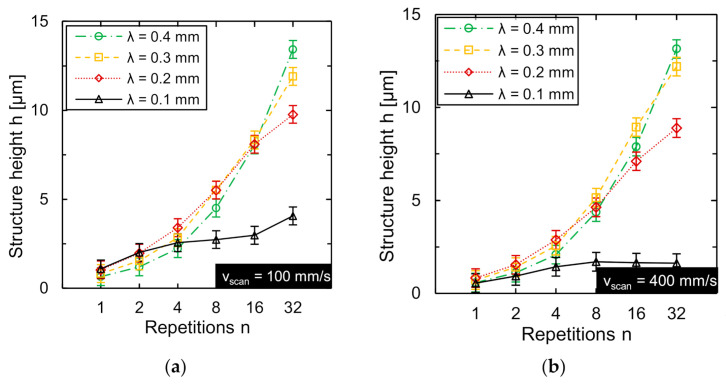
Structure height *h* as function of repetitions *n* for four different wavelengths at scan speeds of (**a**) *v_scan_* = 100 mm/s and (**b**) *v_scan_* = 400 mm/s (*d_L_* = 50 µm, *P_M_* and *P_A_* adapted to *v_scan_*).

Therefore, the influence of repetitions *n* of a processing step on the achieved structure height *h* was investigated. A single track was unidirectionally remelted *n* times with the same modulated laser power signal. The number of passes *n* was investigated in the range from one to thirty-two passes in six steps. Thereby, the number of repetitions was doubled stepwise in each case. The generated structure height after sixty-four passes is already so large that it can no longer be measured using WLI. The dependence of the generated structure height on the number of passes *n* was investigated exemplarily for the wavelength range *λ* = 0.1 mm to *λ* = 0.4 mm in four steps, while the scan speed was investigated in the range from *v_scan_* = 100 mm/s to *v_scan_* = 500 mm/s in five equidistant steps. The process limit was adapted based on the process parameter combination used and the maximum laser power amplitude was used. The dependence of the obtained structure height *h* on the number of passes/repetitions *n* is exemplarily shown for wavelengths *λ* = 0.1–0.4 mm and scan speeds of *v_scan_* = 100 mm/s and *v_scan_* = 400 mm/s (Figure 9).

A significant increase of structure height *h* was achieved by multiple processing. However, at wavelength *λ* = 0.1 mm and scan speed *v_scan_* = 100 mm/s, the increase in structure height is small. Furthermore, it is noteworthy that almost no increase in structure height occurs at *λ* = 0.1 mm and *v_scan_* = 100 mm/s when the number of passes n is greater than four (*n* ≥ 4). At *n* = 1 and *v_scan_* = 100 mm/s, the structure height *h* was approx. 0.86 µm. Processing twice (*n* = 2) increased the structure height to *h* ≈ 1.47 µm. This corresponds to a magnification factor of approx. 1.79. However, at *n* = 32, the structure height was only increased to approx. 2.69 µm. The magnification factor with respect to *n* = 1 is thus only approx. 3.1. At the wavelength *λ* = 0.2 mm, a significant increase in the structure height was observed from *n* = 1 to *n* = 16. After single processing (*n* = 1), the structure height was approx. 0.94 µm. The structure height *h* at *n* = 16 was approx. 7.48 µm and the magnification factor corresponds to approx. 8. The structure height increased further at *n* = 32, but the magnification factor in comparison to *n* ≤ 16 decreases. The achieved structure height was approx. 8.36 µm at a magnification factor of approx. 8.9. When structuring of wavelengths *λ* > 0.2 mm, the achieved structure height was further increased when the number of passes was increased (*n* ≥ 16). For example, at *λ* = 0.4 mm, the structure height *h* ≈ 0.63 µm at *n* = 1 was increased by a factor of approx. 21 to *h* ≈ 13.29 µm at *n* = 32.

The increase of the structure height at small wavelength *λ* = 0.1 mm is comparatively small, while the largest structure height is obtained at the largest investigated wavelength *λ* = 0.4 mm and the most repetitions *n* = 32 (*h* ≈ 12.57 µm). For wavelengths of *λ* > 0.2 mm, the structure height became larger when the number of repetitions was increased at wavelengths *λ* = 0.1 mm or *λ* = 0.4 mm. While the maximum structure height was achieved in the range between *λ* = 0.15 mm and *λ* = 0.25 mm for single processing, the preferred working range for multiple laser processing is at a comparatively large wavelength (e.g., *λ* = 0.4 mm) and a large number of repetitions (e.g., *n* = 32).

**Figure 10:**
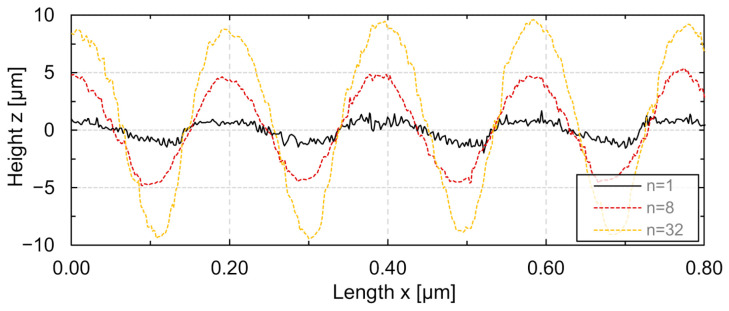
Longitudinal section of single track after surface structuring using *n* = 1, 8, and 32 passes (*d_L_* = 50 µm, *v_scan_* = 400 mm/s, *λ* = 0.2 mm, *P_M_* = 14.5 W and *P_A_* = 5.5 W adapted to *v_scan_*).

An undesirable micro-roughness often remains after single processing on the structured single track. Due to the small laser beam diameter, only a comparatively small amount of material was remelted, so that the initial roughness was relatively large compared to the generated structure height. A reduction of the high frequency structural noise (micro-roughness) was achieved by multiple processing. A change of the structural cross section for an increasing number of repetitions *n* is shown in Figure 10. The initial surface roughness was significantly reduced by multiple processing and is exemplarily shown for *v_scan_* = 400 mm/s and *λ* = 0.2 mm (*n* = 1, *n* = 8, *n* = 32).

### 3.5. Influence of Scan Speed and Number of Passes at Constant Processing Time

An investigation of the obtained structure height at constant processing time *t_B_* was conducted for the range of scan speeds from *v_scan_* = 100 mm/s to *v_scan_* = 500 mm/s. The investigated wavelengths were *λ* = 0.1 mm, *λ* = 0.2 mm, *λ* = 0.3 mm, and *λ* = 0.4 mm. Depending on the scan speed and the number of passes, the processing time for each single track is calculated as follows: *t_B,single track_* = SVL · *v_scan_*^−1^ · *n* (neglecting additional auxiliary times). For example, the generation of a 5 mm long single track with *v_scan_* = 100 mm/s requires *t_B,single track_* = 50 ms. At a scan speed of *v_scan_* = 500 mm/s, the number of repetitions was increased to *n* = 5, so that the processing time remained the same. This was done analogously for all scan speeds.

**Figure 11:**
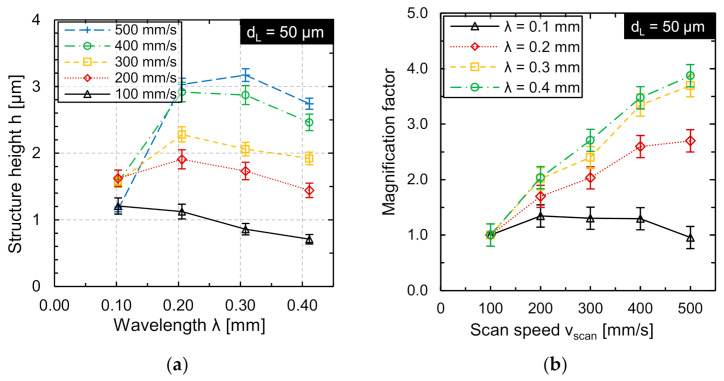
(**a**) Structure height *h* as function of scan speed *v_scan_* and adapted repetitions *n*; (**b**) magnification of structure in comparison to the structure height achieved for the respective wavelength at *v_scan_* = 100 mm/s and *n* = 1 (*d_L_* = 50 µm, *v_scan_* = 200 mm/s, *P_M_* and *P_A_* adapted to *v_scan_*).

In Figure 11a, the achieved structure heights *h* are plotted as function of the wavelength *λ* for different scan speeds *v_scan_* with a corresponding number of repetitions *n*. A maximum structure height of approx. 3.17 µm was achieved at a scan speed of *v_scan_* = 500 mm/s and a wavelength of *λ* = 0.3 mm. The structuring of small wavelengths *λ* = 0.1 mm and large scan speeds *v_scan_* = 400 mm/s and *v_scan_* = 500 mm/s was not meaningful due to the technical limitations of the system technology. A significant increase in structure height was achieved at large wavelengths and high scan speeds. The largest structure height was produced at *v_scan_* = 500 mm/s and *λ* = 0.3 mm (*h* = 3.07 µm).

The magnification factor Δ*h* was calculated as the quotient of structure height *h* (*v_scan_* = *j*·100 mm/s; *n* = *j*) and structure height at *v_scan_* = 100 mm/s and *n* = 1 according to Equation (2):

(2) $$∆h=\frac{h\;\left(v_{scan}=j\cdot 100\;\mathrm{mm}/s;\;n=j\right)\;}{h\;\left(v_{scan}=100\;\mathrm{mm}/s;\;n=1\right)};\;j\in \left[2,\;3,\;4,\;5\right].$$

Figure 11b shows the magnification factor Δ*h* as a function of the examined scan speeds at different wavelengths and for the same processing time. The largest increase in magnification factor occurred at a larger wavelength *λ* = 0.4 mm and was approx. 3.87 at *v_scan_* = 500 mm/s. No significant increase in the magnification factor occurred at a larger scan speed with a corresponding number of repetitions for the wavelength *λ* = 0.1 mm. Overall, the magnification factor significantly decreases for smaller wavelengths.

For structuring at high scan speed and the corresponding number of repetitions, larger structure heights were achieved (exception *λ* = 0.1 mm). This suggests that structuring using high scan speeds has a significant advantage due to the corresponding multiple processing, and thus greater structure heights will be achieved in the same processing time. Additionally, a smoother structure profile was produced at the same time due to multiple processing.

### 3.6. Areal Structuring and Track Offset

In this section, structuring at a constant processing time *t_B,area_* is investigated on the basis of two-dimensional, areal processing. In the previous single track investigations, the track offset was approximately ten times the laser beam diameter to avoid cross-interaction of neighboring tracks. To achieve two-dimensional machining, the track offset is reduced to such an extent that an overlap of the remelt tracks occurs during machining. The achieved structure heights at constant processing time are investigated for scan speeds from 100 to 500 mm/s. The product of scan speed and track offset *dy* remains constant, so that the processing time remains constant (Equation (3)).

(3) $$t_{B,area}=\frac{SVL}{v_{scan}}\cdot \frac{BL}{dy}=\frac{A}{v_{scan}\cdot dy}=const.\;\;\overset{A=const.}{\to }\;v_{scan}\cdot dy=const.$$

The scan speed is varied in the range *v_scan_* = 100 to 500 mm/s in five equidistant steps. The track offset is set to *dy* = 12 µm at the scan speed *v_scan_* = 100 mm/s, so that a significant track overlap is ensured even at the laser power minimum (Figure 4). For higher scan speeds, the track offset is adapted in each case according to Equation (3) and is shown in Table 3. In this case, the examined wavelengths are *λ* = 0.2 mm, *λ* = 0.3 mm, and *λ* = 0.4 mm.

**Figure 12:**
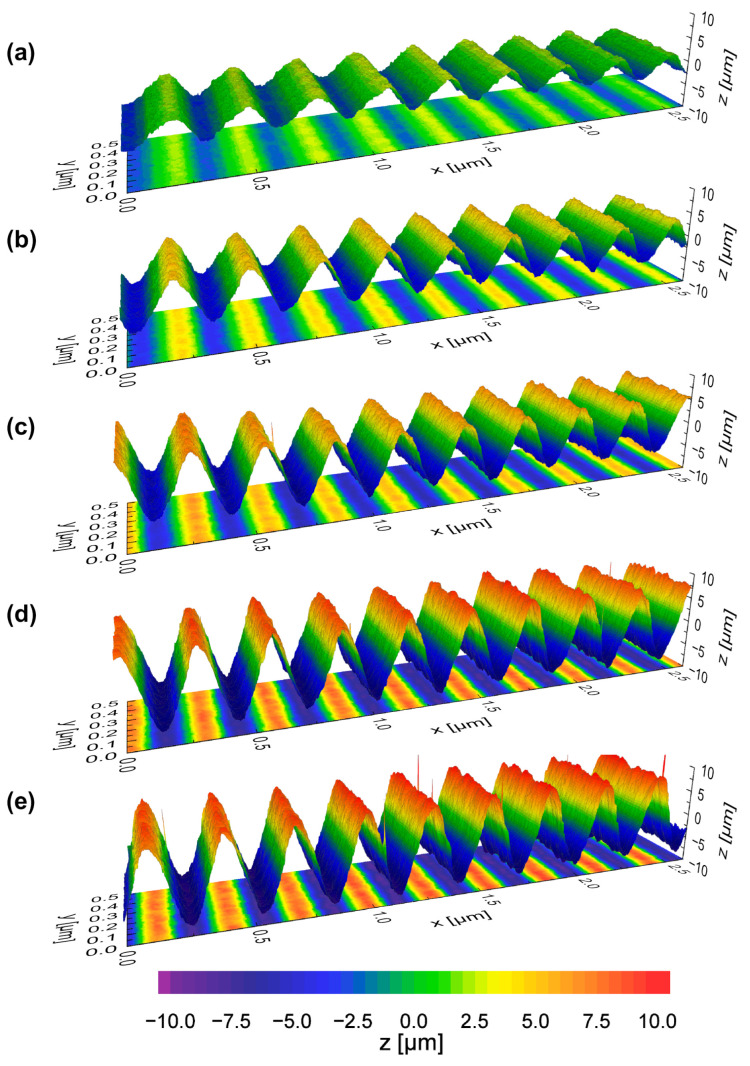
Representative WLI images of surface topographies after areal structuring using (**a**) *v_scan_* = 100 mm/s, *dy* = 12 µm, *n* = 1; (**b**) *v_scan_* = 200 mm/s, *dy* = 6 µm, *n* = 1; (**c**) *v_scan_* = 300 mm/s, *dy* = 4 µm, *n* = 1; (**d**) *v_scan_* = 400 mm/s, *dy* = 3 µm, *n* = 1; (**e**) *v_scan_* = 500 mm/s, *dy* = 2.5 µm, *n* = 1 (*d_L_* = 50 µm, *t_B_* = const., *P_M_* and *P_A_* adapted to *v_scan_*).

First, areal processing was investigated for singular processing. Figure 12 shows representative WLI images of structured surface topographies at different scan speeds and adapted track offsets after *n* = 1 repetition on Ti6Al4V.

**Figure 13:**
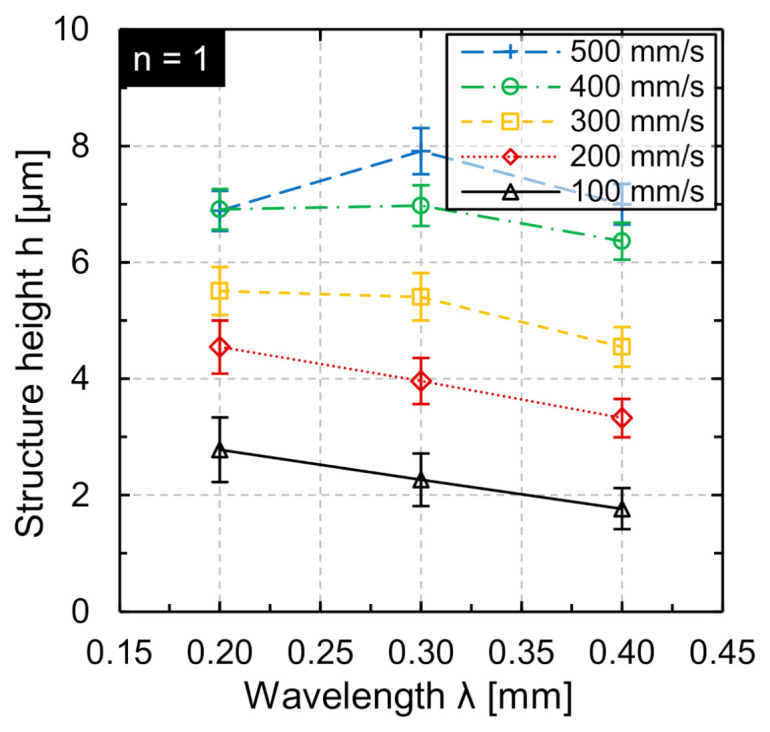
Structure height as function of wavelength λ for different scan speeds *v_scan_* at adapted track offsets *dy* for areal structuring (*d_L_* = 50 µm, *v_scan_* = 200 mm/s, *P_M_* and *P_A_* adapted to *v_scan_*).

In areal processing, larger structure heights were achieved due to the overlap of single tracks (Figure 13). A significant increase in structure height was particularly evident at *v_scan_* = 500 mm/s from *h* = 6.88 µm at *λ* = 0.2 mm to *h* = 7.9 µm at *λ* = 0.3 mm. For a scan speed of *v_scan_* = 100 mm/s, the maximum structure height is approx. 2.78 µm at *λ* = 0.2 mm and decreases to 1.79 µm at *λ* = 0.4 mm. For a scan speed of *v_scan_* = 200 mm/s, the maximum structure height is approx. 5.5 µm at *λ* = 0.2 mm and decreases to approx. 4.55 µm at *λ* = 0.4 mm. At constant processing time, the choice of a high scan speed and multiple processing is beneficial since larger structure heights were produced.

**Figure 14:**
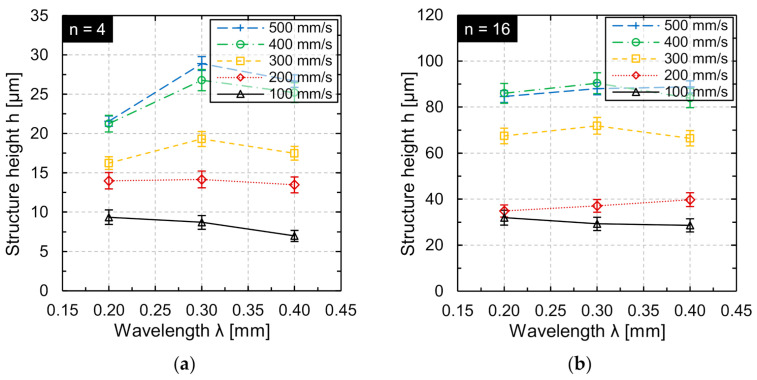
Structure height h in areal structuring for the same processing time as function of wavelength at adapted scan speed and track offset after (**a**) *n* = 4 repetitions and (**b**) *n* = 16 repetitions (*d_L_* = 50 µm, *P_M_* and *P_A_* adapted to *v_scan_*).

In general, a reduction of the track offset *dy* is qualitatively equivalent to multiple machining. Therefore, an investigation of the influence of multiple machining on the structure height was carried out for areal structuring at constant processing time. Using the same process parameter combinations, the number of repetitions was set to *n* = 4 and *n* = 16 for the wavelengths *λ* = 0.2 mm, *λ* = 0.3 mm, and *λ* = 0.4 mm (Figure 14).

In the case of quadruple processing (*n* = 4), the structure height is increased by a factor of 3 to 4 with respect to single processing. For multiple processing *n* = 16, a maximum of approx. 14 times the structure height was produced compared to structuring for *n* = 1. The maximum structure height was generated at *v_scan_* = 500 mm/s, *dy* = 2.4 µm, and *n* = 4 (*h* ≈ 29 µm at *λ* = 0.3 mm). Furthermore, the maximum structure height at *n* = 16 was approx. 90 µm (*v_scan_* = 400 mm/s; *λ* = 0.3 mm; *dy* = 3 µm).

For *n* = 4 as well as for *n* = 16, there was a significant increase in the achieved structure height for increasing scan speed. Furthermore, structuring at *v_scan_* = 500 mm/s was not advantageous compared to structuring at *v_scan_* = 400 mm/s at wavelength *λ* = 0.2 mm as well as *λ* = 0.3 mm. This may be caused by the fact that the efficiency of multiple processing decreases when the number of repetitions is increased or more likely that the modulation frequency at *v_scan_* = 500 mm/s is already limited and results in a reduction of the laser power amplitude. The dependence of the structure height on scan speed at different wavelengths leads to the conclusion that areal processing using high scan speeds and multiple processing steps is beneficial to generated larger structures, particularly for “large” wavelengths. Moreover, for a combination of small wavelengths and many repetitions/remelting cycles, there was no advantage expected in structuring at large scan speeds, since a decrease in wavelength-dependent efficiency has been observed in multiple studies [25,28]. However, even for a wavelength of *λ* = 0.2 mm a structure height up to approx. 85 µm was achieved.

## 4. Discussion

### 4.1. Track Width and Laser Power Amplitude

The results for laser power amplitude show that structure height increases linearly as function of the laser power amplitude. This is in good agreement with experimental results for larger laser beam diameters on the same material [23] and for numerical/experimental results on tool steel H11 [24] or on nickel-based super alloy Inconel 718 [25]. The approximately linear function of remelted area on laser power indicates that the track width is much larger than the remelting depth and leads to an 1D heat conduction approximation as it is common, e.g., for pulsed laser remelting in laser polishing of steel [31], laser micro polishing of Ti6Al4V [32] or in pulsed laser micro melting [33]. Overall, the smaller laser beam diameter leads to significantly smaller volumes of the melt pool and, thus, to comparatively small structure heights of up to 1.5 µm, if no material ablation is aimed for. This is in good agreement with the current understanding of the active process principle [24].

### 4.2. Wavelength

The minimum wavelength is limited due to two main effects. Firstly, the minimum wavelength could not be smaller than the doubled laser beam diameter, since the melt pool surface needs considerable space to be deformed [24]. Secondly, the dynamics of the laser beam source limit the minimum wavelength at a specific wavelength. The modulation frequency *f_Mod_* is the quotient of scan speed *v_scan_* and wavelength *λ* (*f_Mod_* = *v_scan_*·*λ*^−1^). Since this study aimed to investigate high scan speeds and small wavelengths, comparatively high modulation frequencies were reached. In general, a laser beam source requires considerable times for the rise and fall of the laser power. These rise and fall times limit the maximum modulation frequency of the SPI fiber laser to approx. 3 kHz, which is already significantly higher than in previous studies on the WaveShape process [20,23,28]. Nonetheless, if this modulation frequency was exceeded (e.g., *v_scan_* = 400 mm/s, *λ* = 0.1 mm), it led to an undesirable reduction of the achieved structure height-additionally to the existing limitation due to the size of the laser beam diameter. Exceeding the maximum modulation frequency only occurred for few combinations of scan speed and wavelength. Thus, using a laser beam diameter of approx. 50 µm, structures with small wavelengths such as *λ* = 0.1 mm, could be generated and investigated.

Overall, the achieved structure heights achieved in this study are significantly smaller than the structure heights achieved with higher laser power and larger laser beam diameters on Ti6Al4V [23]. Nonetheless, a reproducible micro-structuring with wavelengths significantly smaller than 1 mm was achieved.

### 4.3. Number of Repetitions and Process Time

The investigations showed that multiple processing with the same set of process parameters leads to a significant increase of structure height, which is also in good agreement with previous studies for larger laser beam diameters ([20,23,26,27,28]). However, the increase in structure height is largest for the largest wavelengths. This is presumably a consequence of the remelting process. Similar to laser polishing using continuous wave laser radiation [34,35,36], multiple processing steps lead to a reduction of the height of surface features due to material redistribution. This material redistribution primarily due to capillary forces-counteracts the surface structure formation in the WaveShape process. This process inherent material redistribution is expected to increase with higher number of repetitions, since any additional repetition presumably increases heat accumulation in the processing area, which would lead to larger melt pools. Furthermore, Ukar et al. [34] have shown that the surface roughness reduction in laser polishing/remelting gets more effective for larger surface features. Therefore, the larger the already achieved structure height, the more pronounced the smoothing effect gets due to remelting. Nonetheless, a surprising result of this section is that structure height is almost the same regardless of the scan speed used. Particularly, when multiple processing steps were used at high speed, significantly larger structure heights were achieved. This has not been observed in the Waveshape process and requires further investigations. Particularly, laser beam sources with high beam quality, high laser power, and high modulation frequencies are interesting for further detailed studies. One possible hypothesis is that local heat accumulation leads to higher temperatures in the processing areas and to partial ablation of small amounts of material. Hilton and Nguyen [18], for example, found that local heat accumulation is a considerable factor in the Surfi-Sculpt process and significantly affects the outcome of the structuring process in terms of geometrical dimensions of the generated surface features. Earl et al. [19] hypothesized that vapor pressure (in this case, however, from a keyhole) might also play a crucial role in the Surfi-Sculpt process. Therefore, vaporization might occur, and the correspondingly generated vapor pressure might help increase the structure height as has been hypothesized for Inconel 718 [25], and as has been shown for a hybrid structuring process on Ti6Al4V by Temmler et al. [37].

### 4.4. Areal Structuring and Processing Time

In areal processing, the track distance *dy* is decisive to achieve the interaction of neighboring tracks. The track width as function of laser power was used to determine a suitable minimum track distance to achieve the track overlap along the complete remelted track. A reduction in track offset is similar to multiple processing of single tracks and results in a significant increase of surface structures as has been reported for various materials, laser beam diameters, and scan speeds [20,23,25,26,27,28]. However, the interdependency of track offset *dy*, scan speed *v_scan_*, and number of repetitions *n* is surprising, since it clearly shows that the highest structures were achieved for the largest scan speed (at constant processing time). Additionally, the generated surface structures show a shape deviation from an ideal sinusoidal profile. These shape deviations are similar to those reported and discussed by Oreshkin et al. [29]. Depending on the track offset and the number of repetitions the profile is skewed in or against the scan direction (Figure 12). The difference between the rising and falling flank of the structure is probably caused by differences between heating rates and cooling rates in the WaveShape process. The heating rates are presumably dominated by the time for thermalization of the laser energy as well as scan speed and laser power. Cooling rates are also significantly affected by thermal diffusivity of the material (more than in heating of the material) and the global and local temperature of the surrounding bulk material. Furthermore, the spatial laser power gradient is determined by the laser power amplitude and the wavelength. Moreover, the temporal laser power gradient is affected by the scan speed. In sum, this leads to a complex interdependency of process parameters and thermophysical properties of the material. Typical heating and cooling rates for the WaveShape process using larger laser beam diameters on tool steel can be found in Temmler and Pirch [24].

Finally, Figure 14 shows remarkable results for the Waveshape process. Particularly that structures height between 85 to 90 µm could be achieved for a wavelength of *λ* = 0.2 mm. This is a new dimension for the WaveShape process, since surface features with an aspect ratio close to 1:1 (height:width) were generated. Typical for the WaveShape process are structure heights of approx. 50–100 µm at a wavelength of *λ* ≈ 1.0 mm [20,23,24]. A little drawback for practical use seems the rather long processing time per structured area, which was approx. 22 min/cm^2^. However, if structure height increases even further for higher scan speeds (as the results of this study indicate), a significant decrease in processing time can be expected. Moreover, if smaller aspect ratios are required, e.g., 1:5 or 1:10 as for light guiding surfaces [27], the processing time is significantly smaller at approx. 2–5 min per cm^2^ and might already be applicable for industrial processes such as structuring of tool inserts.

### 4.5. Physical and Technical Limitations

Physical limitations of the Waveshape process presumably result from the time required for thermalization and for heat conduction of the thermalized energy. Usually laser radiation is thermalized on the order of femtoseconds and melting due to heat conduction typically requires up to several picoseconds [38]. In the WaveShape process, melting starts at the surface and travels in the material limited by the speed of sound [39]. Practically, melting speeds of hundreds of meters per second were already measured [40]. The time required for melting is typically approximated by the quotient of melt depth and speed of sound [38]. Thus, a 5 µm deep melt pool requires approx. 1 ns to be generated, if the speed of sound is thought to be on the order of ~5 × 10^3^ m/s. In this case, the maximum modulation frequency would be on the order of approx. 1 GHz. However, as studies for heat accumulation in pulsed laser processing show [41,42], many materials require considerably longer times for cooling than for heating, which makes the remelting process often strongly asymmetrical. Temmler et al. [25], for example, found cooling rates on the order of 10^6^–10^7^ K/s for Inconel 718 (*T* > 1.500 K), while Nüsser [32] found cooling rates on the order of 10^8^ K/s (*T* > 1500 K) in laser micro polishing of Ti6Al4V. This indicates that a physical limit regarding modulation frequencies might be in the range between hundreds of kHz and several tens of MHz. Currently available fiber lasers from renown laser companies achieve maximum modulation frequencies of approx. 50 kHz. These systems or laser beam sources with even higher capabilities in terms of modulation frequency (rise and fall time) are specifically worthwhile for further investigations to increase processing speed and structure height. However, not only the available modulation frequency of the laser beam source might be a bottleneck, but also control and command frequencies of the auxiliary equipment necessary to achieve a controlled, reproducible, and space-resolved control of the laser power signal. High modulation frequencies as well as control and command frequencies might be achieved by external modulators, e.g., acousto-optical or electro-optical modulators. In sum, further investigations on the significance of thermophysical properties such as thermal diffusivity on usable modulation frequencies in the WaveShape process are highly relevant for future applied and scientific studies.

## 5. Concluding Remarks

In this study, surface structuring by laser remelting (WaveShape) using a SPI fiber laser at high scan speeds (*v_scan,max_* = 500 mm/s) and small laser beam diameters (*d_L_* = 50 µm) was investigated for the material Ti6Al4V. The objectives were to achieve larger structure heights in a shorter processing time for small wavelengths. In general, some fundamental characteristics of the WaveShape have been confirmed for smaller laser beam diameters, high scan speeds, and small wavelengths. Firstly, the largest structure heights were achieved at a wavelength of approximately three to four times the laser beam diameter (*n* = 1). Secondly, structure height was significantly increased by multiple processing (single tracks) or choosing adapted track offsets (areal processing). Thirdly, structuring of wavelengths smaller or equal than two times the laser beam diameter is not efficiently possible.

Nonetheless, the study also came to some surprising results. Firstly, structure height is almost the same regardless of the scan speed used. Particularly, when multiple processing steps were used at high scan speed, significantly larger structure heights were achieved. Secondly, the interdependency of track offset *dy*, scan speed *v_scan_*, and number of repetitions *n* was surprising, since it clearly showed that the highest structures were achieved for the largest scan speed (at constant processing time).

**Figure 15:**
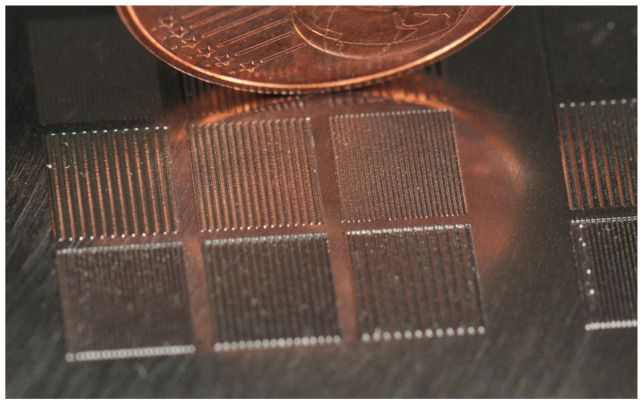
Photo of Ti6Al4V sample with various demo fields (5 × 5 mm^2^) for areal structuring using small laser beam diameters and high scan speeds in the WaveShape process (*d_L_* = 50 µm, *v_scan_* = 200 mm/s, *P_M_* and *P_A_* adapted to *v_scan_*).

Overall, periodic structures with wavelengths smaller than *λ* = 0.5 mm were generated and structure heights larger than a few micrometers were achieved. Furthermore, scan speeds higher than *v_scan_* = 100 mm/s have been systematically investigated and it has been shown that the processing time can be significantly reduced using high scan speeds, adapted laser power modulation, and an adapted number of repetitions. In addition, a structure height to wavelength ratio of approx. 1:1 was achieved for a wavelength of *λ* = 0.2 mm with a processing time of less than 30 s/mm^2^ (Figure 15).

Above all, structuring at high scan speeds has advantages for achieving large structure heights on the one hand and small process times on the other. Both are fundamental advances to further advance surface structuring by laser remelting (WaveShape) towards a broad variety of potential applications.

## Figures and Tables

**Table 1:** Tabular overview of elementary composition (wt%) for Ti6Al4V.

Material/Element ^1^	Ti	Al	V	O	Fe	H	C	N	Rest
Ti6Al4V	89	5.5–6.5	3.5–4.5	0.2	0.3	0.015	0.08	0.05	0.1–0.4

^1^ Based on supplier information.

**Table 2:** Tabular overview of process parameters and range of investigation.

Process Parameter	Range/Value				
Laser beam diameter *d_L_* [µm]	50				
Scan velocity *v_scan_* [mm/s]	100	200	300	400	500
Laser power range *P_min_*–*P_max_* [W]	6–15	7–17	8–19	9–20	10–22
Average laser power *P_M_* [W]	10.9	12.2	13.4	14.5	15.6
Laser power amplitude *P_A_* [W]	4.2	4.65	5.1	5.55	6
Shielding gas (residual oxygen)	Argon (<100 ppm)				

**Table 3:** Tabular overview of process parameters and range of investigation.

Process Parameter	Range/Value				
Scan velocity *v_scan_* [mm/s]	100	200	300	400	500
Track offset *dy* [µm]	12	6	4	3	2.4

## Data Availability

The data presented in this study are available on request from the corresponding author.
